# Gut microbiota-derived metabolites and their importance in neurological disorders

**DOI:** 10.1007/s11033-022-08038-0

**Published:** 2022-11-18

**Authors:** Nicole Mary Swer, B S Venkidesh, Thokur Sreepathy Murali, Kamalesh Dattaram Mumbrekar

**Affiliations:** 1 Manipal School of Life Sciences, Manipal Academy of Higher Education, Manipal, 576104 India; 2 Department of Radiation Biology & Toxicology, Manipal School of Life Sciences, Manipal Academy of Higher Education, Manipal, 576104 India; 3 Department of Biotechnology, Manipal School of Life Sciences, Manipal Academy of Higher Education, Manipal, 576104 India

**Keywords:** Gut–brain axis, Gut-derived metabolites, Gut microbiome, Gut dysbiosis, Neurodegenerative disorders, Alzheimer’s disorder, Short-chain fatty acids

## Abstract

Microbial-derived metabolites are the intermediate or end products of bacterial digestion. They are one of the most important molecules for the gut to connect with the brain. Depending on the levels of specific metabolites produced in the host, it can exert beneficial or detrimental effects on the brain and have been linked to several neurodegenerative and neuropsychiatric disorders. However, the underlying mechanisms remain largely unexplored. Insight into these mechanisms could reveal new pathways or targets, resulting in novel treatment approaches targeting neurodegenerative diseases. We have reviewed selected metabolites, including short-chain fatty acids, aromatic amino acids, trimethylamine-N-oxide,  urolithin A, anthocyanins, equols, imidazole, and propionate to highlight their mechanism of action, underlying role in maintaining intestinal homeostasis and regulating neuro-immunoendocrine function. Further discussed on  how altered metabolite levels can influence the gut–brain axis could lead to new prevention strategies or novel treatment approaches to neural disorders.

## Introduction

The gastrointestinal tract (GIT) hosts more than 100 trillion microbes that together harbour more than 3 million genes [1]; however, the predominant bacteria consist mainly of phyla Firmicutes, Bacteroidetes, Actinobacteria, Proteobacteria, and Verrucomicrobia [2]. The inhabitant bacteria in the GIT carry out an assortment of activities that benefit the host, including the digestion of vitamins, and metabolites (bile acids, amino acids, lipids), regulating pH change and peptide production, and influencing cell signalling pathways. Some of these metabolites are commonly altered due to environmental factors and have further been correlated with neurodegenerative symptoms [3].

Though the microbiota composition is shaped before birth, factors such as the birthing method, feeding method, age, diet, geographic location, genetics, and physical activity determine the core microbiome of an individual [4]. The human microbiome project has shown that human microbiota is more similar across individuals than across body sites and is unique to specific organs [5]. Further, the gut microbiota composition can be altered by factors like pH, bacterial load, oxygen, medicines like antibiotics, and supplements like prebiotics and probiotics [6]. Deviations from normal gut microbiome composition can lead to dysbiosis that can prove detrimental to an individual’s health. Further, it can also act as a biomarker for exposure or treatment response [7]. The microbiota gut-brain axis (MGBA) is a term used to describe the link between the gut and the brain involving the interplay of several systems, mainly the autonomic, central, and enteric nervous systems and the hypothalamic-pituitary axis. Several mechanisms are implicated in MGBA signalling, including the involvement of the vagus nerve, the immune system, vitamins, and bacterial metabolites.

Gut metabolites are the intermediates or end products of bacterial metabolic processes. Some important metabolites produced include aromatic amino acids and short-chain fatty acids (SCFAs) derived from dietary sources such as fibres and fruits; endogenous metabolites derived from bile acids and cholesterol. Their primary signalling pathway is through the vagal and spinal nerves of the autonomic nervous system (ANS). These metabolites also can enter the bloodstream; several of them can permeate the blood–brain barrier (BBB) and influence regulatory mechanisms in the central nervous system (CNS) [8]. The ANS also controls gut permeability, motility, and immuno-modulatory mechanisms, determining the bacterial composition and the metabolites produced. These metabolites have been linked to neurodegenerative, neuroinflammatory, and neuropsychiatric disorders such as Alzheimer’s disease (AD), Parkinson’s disease (PD), and autism spectrum disorders (ASD) [9–11]. They have been found to alleviate/exacerbate various neuronal symptoms. Further insight into their underlying mechanisms could shed light on the neuromodulatory pathways and lead to practical novel therapeutic approaches. This review briefly summarizes the mechanisms of various metabolites like SCFAs, aromatic amino acids, and other less explored metabolites and their effects on brain health and neural activities.

### Gut-associated neurological disorders

Neurological disorders are characterized by a progressive decrease in nervous system function. Gut microbiome changes in humans have been reported to be one of the many causes of various- psychological and neurological conditions [12]. Using specific pathogen-free (SPF) or germ-free (GF) murine models, researchers hope to unravel the molecular pathways from the gut to the brain [13, 14]. Cross-sectional studies have found that the bacterial taxa *Escherichia* and *Shigella*, which are associated with causing inflammation, are elevated in faecal samples from Alzheimer’s patients compared to healthy people [15]. Additionally, another study showed AD progression might not affect the brain directly but through amyloid deposition in the APP^swe^/PS1^∆E9^ transgenic mice intestine, where they found altered gut microbiome, lowered SCFAs, and altered metabolic pathways [16]. It has been reported that alteration in the composition of bacteria-producing metabolites may initiate and sustain the pathogenic processes of neurodegeneration [17]. So, dysbiosis of the gut contributes to neuroinflammation and injury, which can result in neurodegeneration, key factors associated with AD. Similarly, Parkinson’s disease (PD) is another motor-impaired disorder. Preclinical research suggests that gut microbiota can enhance the aggregation of alpha-synuclein, hamper the elimination process [18], and via the vagus nerve, these proteins can reach from the gut to the brain [19, 20]. The alpha-synuclein can aggregate in various neurons to form Lewy bodies and Lewy neurites, leading to disease pathology. In another study, researchers orally introduced bacterial-derived SCFAs to alpha-synuclein overexpressing mice to test the hypothesis that SCFAs induced alpha-synuclein production in the brain and showed a positive association between SCFAs and PD [21].

In addition, microbial metabolites have been associated with neurological disorders like multiple sclerosis (MS), dementia, and epilepsy [22]. A study comparing the microbiome of 71 untreated MS patients to healthy controls reported a significant increase in *Akkermansia muciniphila* and *Acinetobacter calcoaceticus,* which are associated with MS [23]. Further, the transplantation of gut microbiota of MS patients into germ-free mice indicates the potentially causal role of gut microbiota in MS [20, 21]. These findings suggest that gut microbiota could be linked to the increased susceptibility of MS in humans.

### Microbe-derived metabolites are linked to the MGBA

**Fig. 1:**
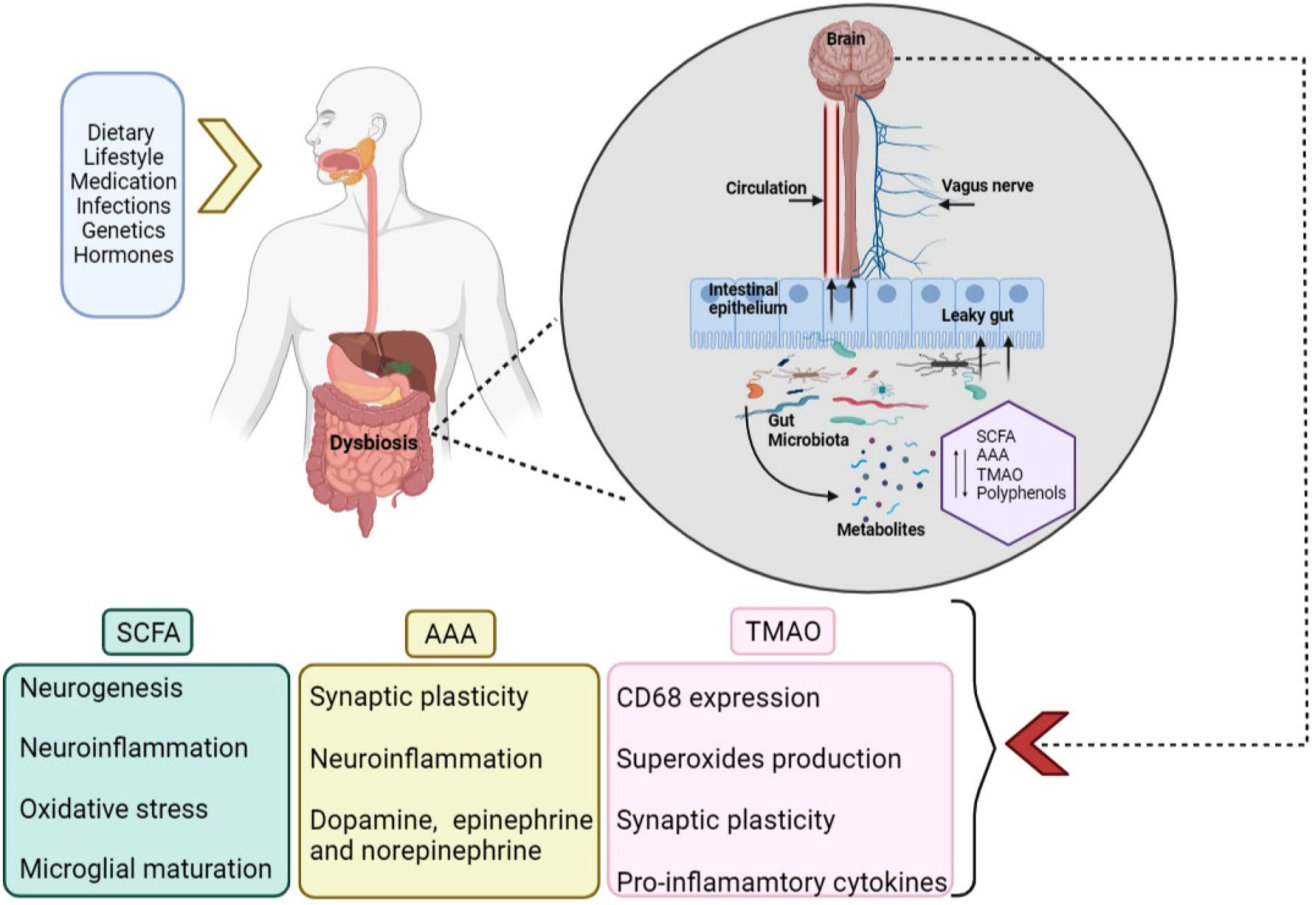
Gut bacteria-derived metabolites and their neuronal effects. Both extrinsic and intrinsic factors can cause dysbiosis of the gut, characterised by increased gut permeability or “leaky gut” and shifts in bacterial taxonomy groups, leading to altered levels of various metabolites. These metabolites can enter the bloodstream and get transported through the ANS to the CNS to alter neuronal activities and modulate the expression of neurotransmitters.

The gut microbiota produces a variety of metabolites that have a diverse array of functions and play a significant role in signalling pathways in the MGBA (Fig. 1). Among these, metabolites like short-chain fatty acids, aromatic amino acids, and trimethylamine-N-oxide, which could influence brain function, have gained wide attention (Table 1). Gut microbiota-derived metabolites can enter host circulation through the gut barrier and can be beneficial or toxic. However, the effects of metabolites are influenced by the epithelial barrier intactness, dietary components like fibres and fruits, geographic location, and ethnicity to which the host belongs [24].

**Table 1:** Gut microbiota-derived metabolites that have been shown to exert effects on the brain

Name of metabolite		Dietary sources of the metabolite	Gut microbiota	Effects of metabolite on brain		References
				Beneficial effects	Detrimental effects	
Short chain fatty acids	Butyric acid (BA)	Almonds, chickpeas, apples, garlic, soy, cheese, and butter	*Firmicutes, Eubacterium rectale, Clostridium leptum, Eubacterium hallii*	Alleviate ASD-like symptoms. Reduces cognitive impairment, improves memory and learning, and enhance neuroprotection. Improves associative memory in advanced stages of AD.		[75] [76] [24]
	Propionic acid (PA)	Processed fruit and vegetable products, dairy eggs processed meats	*Phascolarctobacterium succinatutens, Bacteroides* spp., *Dialister* spp., *Megasphaera elsdenii*	Ameliorate motor and non-motor defects in PD patients.	Role in neurotoxicity by damaging mitochondrial DNA.	[21] [77] [78]
	Acetic acid (AA)	Processed meat, and smoked/frozen fish, vinegar, and alcoholic beverages	*Bifidobacterium bifidum, Bifidobacterium breve, Akkermansia muciniphila*	Ameliorate motor and non-motor defects in PD patients.		[79] [80]
AAM (amino acid metabolites) AAM—TRYP6 metabolites	Taurine	Meat, fish, milk dairy products	*Bacteroides xylanisolvens, Alistipes finegoldii*	Enhances long-term memory. Has anti-oxidant properties.		[81] [82]
	Gamma-Aminobutyric Acid (GABA)	Kimchi, miso, tempeh, fermented foods	*Lactobacillus brevis, Bifidobacterium, Bacteroides*	Protects from neurodegeneration. Reduced levels in ASD patients.		[83] [84]
	Serotonin	Seeds, Eggs, Salmon, Spinach	*Candida, Pseudomonas Streptococcus, Enterococcus, Escherichia*		Decreased levels linked to AD.	[85]
	Kynurenine	Fresh potatoes, sweet potatoes, crisps, flour	*Burkholderia, Pseudomonas, Streptomyces, Bacillus*	Amelioration of neurotoxic effects as NMDA receptor antagonist.	Elevated levels linked with cognitive impairments.	[86–88]
	Indole	Brussels sprouts, cabbage, collards, cauliflower, kale, mustard greens	*Edwardsiella, Shigella, Bacteroides, Desulfitobacterium, Citrobacter, ClostridiumXIX, Escherichia, Providencia*		It is associated with impaired motor function, depression, and anxiety Can invoke hypotension, loss of righting reflex, and a reversible comatose state.	[89] [58]
	Quinolinate	Potatoes, processed meat, processed vegetables, basil, dairy products	*Bacillus, Klebsiella, Burkholderia*		Shows excitotoxic properties as NMDA receptor agonist Promotes neurodegeneration and neurotoxicity.	[86] [87]
	Indole Propionic Acid (IPA)	Pomegranates, sprouted beans, kimchi, sauerkraut, pickles, kefir	*Proteus, Clostridium, Escherichia*	Acts as a neuroprotectant by ameliorating ROS Can reduce neuroinflammation Beneficial anti-oxidative properties for brain functions.		[90] [52] [91]
	Indole Acetic Acid (IAA)	Garlic, cinnamon, quinoa, tomatoes, chickpeas	*Staphylococcus, Ralstonia, Bacillus, Klebsiella*	Suppresses pro-inflammatory cytokine production by macrophages Attenuates neuroinflammation in microglial cells in culture		[92] [93]
Trimethylamine N-Oxide (TMAO)		Tomatoes, soybeans, beef, oranges, peanuts, pears, peas	*Desulfovibrio, Prevotella, Mitsuokella, Methanobrevibacter smithii, Fusobacterium*	In mice, a lipophilic derivative of TMAO improved neurological functions by preventing NSC-34 motor neuron-like cells and primary mouse astrocytes from dying due to endothelial reticulum stress.	Promotes neuronal senescence in hippocampal regions and cognitive impairment by increasing oxidative stress Higher concentrations in cognitive and pathophysiological deterioration TMAO disrupts the blood–brain barrier by lowering the expression of tight junction proteins such as claudin-5 and tight junction protein-1 (ZO-1)	[94] [95] [63] [96] [97]
Other metabolites	Urolithin A	Blackberries, Pomegranate, Strawberries, nuts.	*Gordonibacter pamelaeae* and *Gordonibacter urolithinfaciens*	Improved cognitive functions, inhibited neural apoptosis, induced neurogenesis, and decreased the pro-inflammatory cytokines IL-1β and TNF-α in the cortex and hippocampus		[66]
	Anthocyanins	Berries, grapes, plums, and other foods containing high natural colorants	*Bifidobacterium* and *Lactobacillus* species	Reduced neural apoptosis in an APP/PS1 transgenic mouse model of AD		[68]
	Equols (EQ)	Soy food	*Lactobacillus* sp, *Eggerthella* sp, *Clostridium* sp, *Bifidobacterium* sp, family Coriobacteriaceae	Permeate the blood–brain barrier and reduce the production of pro-inflammatory cytokines hence preventing neuroinflammation		[69]

Amino acids also can undergo microbial fermentation to yield ammonia, phenol, and indole. Signalling molecules derived from tryptophan, like serotonin (5-HT), is responsible for regulating mood, behaviour, sleep, and appetite [25]. 5-HT binds to aryl hydrocarbon receptors (AhR) on astrocytes and can activate microglia modulating neuroinflammation. Their role in depressive disorders and anxiety is being explored [26, 27]. Indole is a tryptophan derivative with neuroactive properties and is reported to cross the blood–brain barrier to regulate the levels in the brain [28]. Neurotransmitters like dopamine are produced from bacteria like *Escherichia,* which metabolizes phenylalanine, an aromatic amino acid. Another metabolite, choline, is essential for neurotransmission and methylation and is metabolized to Trimethylamine N-oxide (TMAO), which is involved in the MGBA and can be detected in the CNS [29].

Microglial activation can be influenced by the circulation of microbe-derived neurotransmitters such as acetylcholine (*Lactobacillus*), gamma-aminobutyric acid (GABA) (*Bifidobacteria *and* Lactobacillus*), and serotonin (*Enterococcus *and* Streptococcus*). According to studies, the gut produces 90% of the serotonin required to regulate mood, behavior, sleep, and other processes in the CNS and GI tract. Serotonin binding to 5-HT receptors on microglia causes the release of cytokine-carrying exosomes, offering yet another pathway for gut-induced neuroinflammation control [30].

The effects of SCFAs have recently been extended to microglia. The immature genetic and morphological phenotype of microglia in GF mice was recovered by supplementing drinking water with three major SCFAs (acetic acid, propionic acid, and butyric acid). Microglial dysfunction is assumed to play a role in the progression of neurological disorders, including ASD and PD. Once in the bloodstream, these chemicals trigger a pro-inflammatory immune response mediated by peripheral T cells and macrophages, compromising the BBB’s integrity [31]. The microbial-derived metabolites are also crucial for neuronal homeostasis and controlling inflammation [32]. Rothhammer et al. [33] highlighted the role of metabolites in regulating the microglia and astrocytes in the CNS, which plays a role in neuroinflammation and neurodegeneration. Increased microglial activation and generation of pro-inflammatory cytokines in the brain may be due to increased circulation of BBB-permeable pro-inflammatory cytokines.

**Fig. 2:**
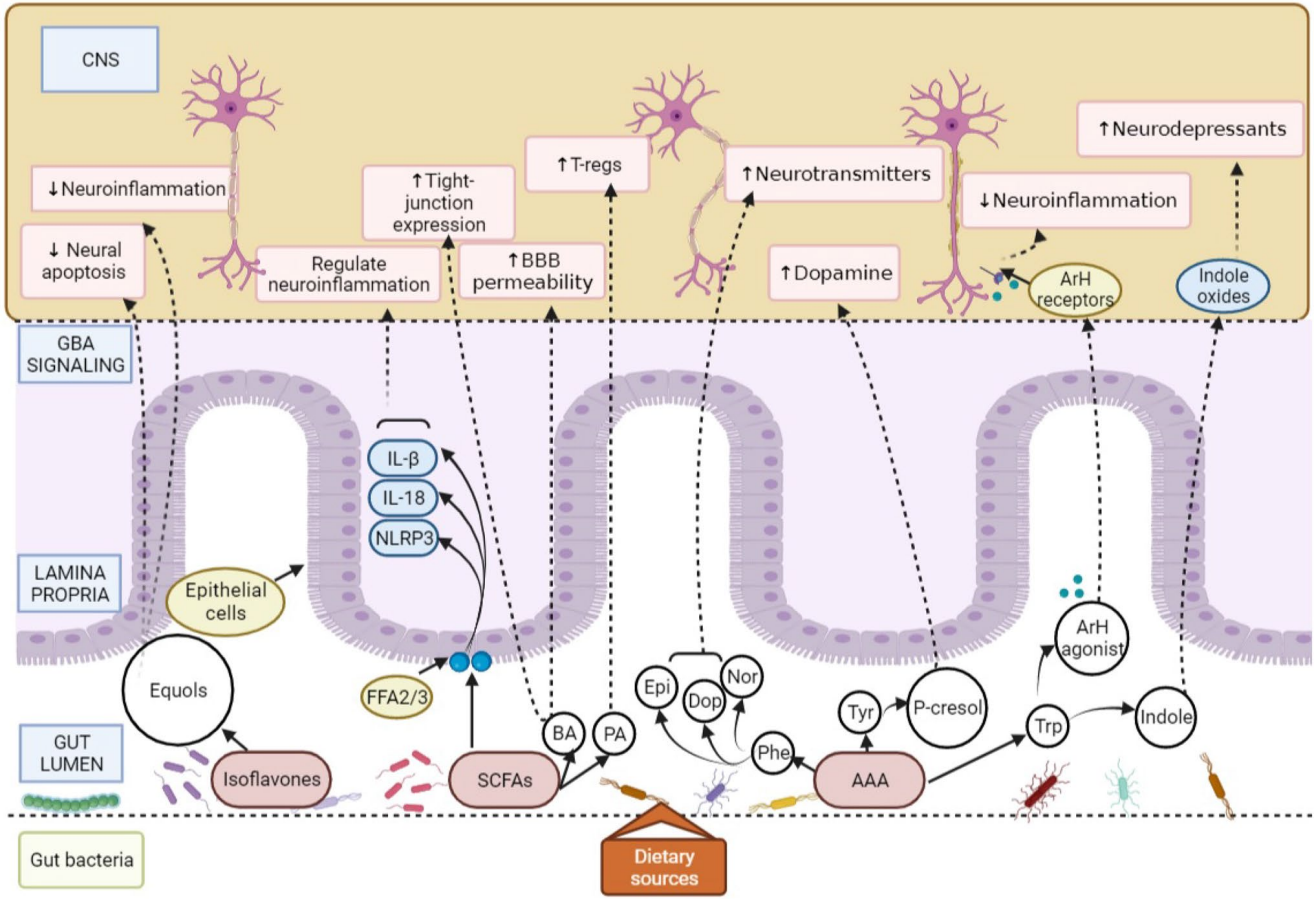
Mechanism of action of gut microbiota-derived metabolites on the CNS. The gut microbiota composition varies across the intestinal lumen and depends on several factors, including dietary sources. The illustration depicts a few relevant metabolites like isoflavones, SCFAs, and AAAs that serve as precursors for secondary metabolites like equols, propionate, butyrate, and neurotransmitters like epinephrine and dopamine. These metabolites participate in MGBA signaling, binding to receptors or agonists and crossing the epithelial barrier to exert their effects in the CNS. Here, they influence several neural functions that can be detrimental or beneficial to the host (↑ increase, ↓decrease). *SCFAs* short chain fatty acids, *AAA* aromatic amino acids, *FFA2/3* Free fatty acid receptors 2 and 3, *BA* butyrate, *PA* propionate, *Epi* epinephrine, *Dop* dopamine, *Nor* norepinephrine, *Phe* phenylalanine, *Tyr* tyrosine, *Trp* tryptophan, *ArH* aryl hydrocarbon, *BBB* blood brain barrier

Alteration of microbial-derived metabolites by oral administration of *Bacteroides fragilis* to the maternal immune activation (MIA) C57BL/6N mouse model that exhibited symptoms relevant to ASD, ameliorated communicative defects and sensorimotor defects; decreased anxiety-like behaviour [22]. These observations indicate that the microbial-derived metabolites influence diverse signalling pathways that control brain functions (Fig. 2). They have been considered promising candidates for ameliorating neurodegenerative disorders. However, mechanisms of action for most of these metabolites remain to be investigated.

#### Short chain fatty acids (SCFAs)

SCFAs are gut microbiota-derived metabolites produced by fermentation of non-digestible carbohydrates. The major SCFAs produced in comparatively large ratios are acetate, propionate, and butyrate, whereas other SCFAs like isovalerate and isobutyrate are produced in trace amounts [34, 35]. SCFAs play a significant role in maintaining the intestinal barrier, regulating inflammatory response, and mucus production [36]. SCFAs are also natural ligands for receptors like free fatty acid receptors 2 and 3 (FFA2/FFA3) attached to immune cells and enteroendocrine cells [37], allowing them to modulate the levels of several pro-inflammatory cytokines that can induce neuroinflammation. Further, SCFAs, when coupled with G-protein receptors, can modulate the production of pro-inflammatory cytokines, activate inflammasome pathways like the NLRP3 pathway, and cause inflammation [24]. SCFAs are also one of the primary metabolites implicated in the MGBA, and several studies have highlighted their importance in influencing several other body organs, including the brain [38].

Clear evidence of uptake of SCFAs by the brain was demonstrated by Oldendorf et al. [39], where SCFAs were injected into the common carotid artery of Wistar rats, and all the three significant SCFAs were detected in the Cerebrospinal fluid (CSF) in varying ratios. Supplementation with SCFAs has altered several functions related to the brain. Li et al. [40] demonstrated that SCFAs prevented neuroinflammation in fructose-fed mice by reducing the pro-inflammatory cytokines like IL-1β, TNF-α, IL-6 mRNA levels, and microglial activation. One of the major systems involved in the GBA- the Hypothalamic Pituitary Axis, a core stress axis, is also affected by SCFAs. Various studies have correlated altered gut microbiota composition with varying stress levels. One study demonstrated that when mice were administered a mixture of sodium acetate, propionate, and sodium butyrate, they exhibited altered anhedonia and increased stress-responsiveness [41].

Due to the increasing evidence supporting SCFA’s role in affecting neural functions, they are being investigated for their role in neurodegenerative disorders. Marrizzoni et al. [42] reported a link between SCFAs (acetate and valerate) and endothelial damage, which correlates to the regulation of the BBB and, in turn, contributes to AD pathogenesis. A critical activity of SCFAs is their ability to act as histone deacetylase inhibitors (HDACi). They can alter histone acetylation during memory formation. Considering that SCFAs can act as HDACi, it has been hypothesized that they can be used as a therapeutic in treating AD to correct aberrant histone acetylation, which is associated with the pathogenesis of AD [42].

Further investigation could reveal their more profound role in learning and memory. Evidence shows that SCFAs are altered in individuals with ASD. A study on GI disorders and pathogenesis of ASD reported decreased levels of faecal acetic acid, butyrate, and propionic acid comparatively increased levels of valeric acids in ASD subjects [43]. In a study conducted by Zeng et al. [44], an analysis of faecal samples of Amyotrophic Lateral Sclerosis (ALS) patients revealed an increase in butyrate levels to reduce the accumulation of abnormal proteins. Similarly, in a G93A transgenic mice model of ALS, animals fed with butyrate were shown to have improved gut homeostasis and a longer life span [45]. However, the causal role between ALS and metabolites is still not well established. Further investigations need to be carried out to understand whether the alteration in levels of various gut metabolites is the cause or affects ALS.

Chen et al. [46] demonstrated that supplementing butyric acid to rat middle cerebral artery occlusion models reduced neurological impairment, lipid levels, and risk of thrombosis. Butyric acid treatment has been shown to increase the microbial alpha diversity with an increase in *Lactobacillus*, which has been reported to have beneficial effects like preventing neuronal cell death, reducing oxidative stress, and repairing abnormal neurobehavior [46]. Supplementing sodium butyrate to rats with ischemic brain showed upregulated Brain-Derived Neurotrophic Factor (BDNF) levels, increased neurogenesis, and neural proliferation [47] played a role in the memory process [48].

In another study, Erny et al. [49] demonstrated that the microglia of GF mice showed defects that were partially restored into a mature phenotype when recolonized with complex gut microbiota or fed with SCFA (a mixture of acetate, butyrate, and propionate). They can also affect brain neurochemistry by regulating the expression of 5-HT enzymes, which are serotonin precursors. Increased SCFA levels were also associated with increased growth of human progenitor cells, implying that they could influence early neural development. Further, physiologically relevant levels of acetate, propionate, and butyrate enhanced the growth rate of cells and induced mitosis [50]. Studies have also demonstrated the ability of SCFAs to affect the sleep and appetite signals of the brain by modulating neurons like orexigenic neurons and receptors such as the ghrelin receptor [51]. This mechanism is revealed to be brought about by coupling SCFAs to FFA2/3 receptors. Overall, SCFAs tend to regulate GI function, circadian rhythm, and other neuroimmune functions; however, the studies mentioned above have indicated a role in lowering neuroinflammation, regulating the blood–brain barrier, and, most importantly, ameliorating memory loss associated with neuroinflammatory disorders.

#### Aromatic amino acid (AAA) metabolites

All three aromatic amino acids—phenylalanine, tyrosine, and tryptophan, play a significant role in the MGBA and are a product of gut microbial metabolism. These three amino acids serve as precursors to several secondary metabolites, some of which act as neurotransmitters and play a role in brain health [52]. For example, phenylalanine is the starting product that produces dopamine, norepinephrine, and epinephrine, whereas tryptophan is the precursor to 5-HT and serotonin, vitamin B3, and redox co-factors like NAD(P)^+^. Even kynurenine pathway metabolites like indole and its derivatives are produced from the AAA metabolism [53]. Tryptophan metabolite- kynurenine was shown to reduce the activity of Natural Killer (NK) and dendritic cells in the CNS, and accumulation of this metabolite in the brain is associated with depression and schizophrenia [50]. Phenylalanine affects neurotransmission by producing amino acids like glutamate and aspartate, which act as excitatory neurotransmitters [53]. Another amino acid, glycine, also made from phenylalanine, is an inhibitory neurotransmitter [54]. Serotonin is a neurotransmitter that regulates gastrointestinal secretion motility and pain perception, regulates mood and cognition behaviour in the brain, and is produced by the gut mucosal enterochromaffin cells [25]. These studies suggest that AAAs should be investigated further for their role in influencing neural disorders.

#### Tryptophan metabolites

The gut microbiota plays an essential role in tryptophan metabolism, a precursor to serotonin production. More than half of serotonin is produced in the gut and is responsible for activating several receptors on the enteric neurons and immune cells. Serotonin also binds to 5-HT receptors in the microglia and mediates neuroinflammation in the CNS. Furthermore, tryptophan gives rise to bacterial metabolites that can control CNS inflammation through receptors like the AhR [55]. Tryptophan metabolites have been shown to modulate metalloproteinases that control the degradation of amyloid-beta (Aβ)﻿peptides through AhR in the CNS. A recent review indicated that 5-hydroxy indole acetic acid and kynurenic acid could prevent the formation of Aβ plaques [10]. Tryptophan levels can also reduce anxiety-like behaviour. For example, when restoring microbiota to germ-free mice, they exhibited reduced anxiety levels, 5-HT, and 5-Hydroxindoleacetic acid levels compared to conventionally colonized mice [25]. In mice, a tryptophan-rich diet slows brain aging by decreasing oxidative stress and inflammation by regulating AMP-activated protein kinase (AMPK) and Nf-kB pathways [56]. In a high level of cognitive strain, the injection of 5-HT2A/2C or 5-HT4 receptor agonists or 5-HT1A, 5HT3, and 5-HT1B receptor antagonists avoids memory loss and improves learning [57]. This indicates the beneficial effects of tryptophan in modulating neuroinflammation and neurogenesis.

Indole, another metabolite produced from precursor tryptophan by an enzyme tryptophanase A (*tnA*), is said to induce depression and impact normal emotional behavior. Treating rats with indole led to the accumulation of its oxidized derivatives in the brain, negatively affecting the rats by reducing motor activity and inducing a higher anxiety-like behaviour [58]. P-cresol, derived from tyrosine, plays a major role in several neurodegenerative disorders like ASD. In a BTBR ASD mice model, increased doses of P-cresol were said to worsen certain symptoms of ASD and activate dopamine levels in specific brain regions like the amygdala [59]. Another study reported how p-cresol inhibited the formation of oligodendrocytes, which might hamper the formation of myelinated neurons in the mice CNS [60]. As the studies reported, microbial-derived amino acids and tryptophan metabolites tend to induce oxidative stress and inflammation, reducing motor activity and inducing a higher anxiety-like behavior. Studies showed that 5-hydroxy indole acetic acid and kynurenic acid could prevent the formation of Aβ﻿-peptide plaques [10]. Recent reports suggest that P-cresol sulfate and 4-ethyl phenyl sulfate, two microbiota-derived host metabolites, are elevated in ASD patients and animal models such as BTBR and MIA mice [59]. However, further research is required to understand the link between these metabolites and ASD. Overall, the studies indicate the potential beneficial effects of tryptophan in modulating neuroinflammation and neurogenesis.

#### Trimethylamine N-oxide (TMAO)

TMAO is another microbe-derived molecule produced by several bacterial species from dietary constituents such as choline and L-choline. They can be detected in the CSF, implying that it is involved in the CNS and has been investigated for its pathophysiological role in neurodegenerative disorders [29]. TMAO has been implicated in numeral cerebrovascular diseases like atherosclerosis [61], a known risk factor for dementia. It also induces CD68 expression, which is a marker associated with dementia. It causes neuronal aging, disrupts mitochondrial functions, and increases oxidative stress.

A study conducted by Vogt et al. [29] to investigate the role of TMAO in AD showed that elevated levels of TMAO were detected in CSF collected from individuals with clinical AD and individuals with mild cognitive impairment (MCI) when compared to cognitively unimpaired individuals. The elevated levels were also linked to phosphorylated tau and Aβ. Further studies to understand the specific role of TMAO in neurodegenerative diseases like Parkinson’s could shed further light on the pathophysiology of the disease. TMAO can induce brain aging and cognitive dysfunction. The link between TMAO and brain aging was analyzed by Li et al. [62], who showed that plasma levels of TMAO were increased in senescence-accelerated prone mouse strain 8 (SAMP8) compared to control mice. These mice also exhibited cognitive dysfunction with more senescent cells in the hippocampal CA3 region. The observed neuronal damage and reduced synaptic plasticity were associated with increased superoxide production and impaired mitochondria. These changes mainly involved mammalian target of rapamycin (mTOR) signaling. This activity relates to spatial learning, object recognition, and memory by regulating receptors like N-Methyl D-Aspartate receptor subunit 1.

In an AD (3xTg-AD) model, increased levels of TMAO were associated with altered presynaptic and reduced postsynaptic receptor expression, which was brought about by the stress signaling pathway [63]. Brunt et al. [64] provided evidence linking TMAO levels, neuroinflammation, and cognitive decline. The study comparing mice of different ages (27 months vs. 6 months) showed that with aging, TMAO concentrations increased, which correlated to higher pro-inflammatory cytokines and astrocyte activation. Additionally, when 6-months old mice were supplemented with TMAO, they performed poorly in the novel object recognition test and exhibited astrocyte activation. A reduction of TMAO levels in APP/PS1 mice led to a decrease in cognitive deterioration, Aβ and beta-related enzymes like beta-secretase in the hippocampus, and hippocampal neuroinflammation [65]. Overall, studies indicate that TMAO is linked to the development of age-related cognitive deterioration and is associated with AD, diabetes mellitus, and cardiovascular illnesses. However,the mechanisms leading to observed effects remain unknown.

### Other metabolites

Urolithin A is a significant gut-derived metabolite derived from ellagic acid, whose sources include pomegranates, berries, and nuts. In-vivo and in-vitro studies have demonstrated the antioxidant and anti-inflammatory effects of these metabolites; however, the underlying molecular mechanisms still need to be elaborated upon. Gong et al. [66] investigated the effects of urolithin A in alleviating AD symptoms and showed improved cognitive functions, inhibited neural apoptosis, induced neurogenesis, and decreased the pro-inflammatory cytokines IL-1β and TNF-α in the mice cortex and hippocampus. Thus, Urolithin A is a promising therapeutic target for improving the pathophysiology of AD.

Anthocyanins are microbial-derived metabolites whose dietary sources include berries, grapes, plums, and other foods containing high natural colorants. They prevent oxidative stress, have neuroprotective effects, reduce neuroinflammation and regulate cell signaling pathways [67]. Ali et al. [68] described the role of Korean black bean anthocyanin in reducing neural apoptosis in an APP/PS1 transgenic mouse model of AD. Several *Bifidobacterium* species and *Lactobacillus* produce metabolites like GABA and inhibitory neurotransmitters.

Equols (EQ) are gut-derived metabolites obtained by metabolizing isoflavones. They protect microglia against oxidative stress, prevent neural apoptosis, and induce neural generation. They have also been extensively studied for their bioactive role in selected bone and cardiovascular disorders. Johnson et al. [69] demonstrated that EQs could permeate the blood–brain barrier and reduce the production of pro-inflammatory cytokines, preventing neuroinflammation. In another study, SOD1-Tg mice were administered with A*kkermansia muciniphila*, which resulted in the accumulation of nicotinamide in the CNS, ameliorating several symptoms of ALS, improved motor symptoms, and altered gene expression levels in the spinal cord [70].

The microbial metabolite imidazole propionate has been found to disrupt insulin signaling and lead to type II diabetes, which can negatively affect cognitive functions of the brain and has been known to be a major risk factor in the development of Alzheimer’s [71, 72]. Other metabolites such as dihydrosphingosine, phytosphingosine, inosine, and hypoxanthine have also alleviated AD symptoms. When Xanthoceraside, a compound proven to have anti-Alzheimer’s activity, was administered, altered levels of these metabolites accompanied by a change in the gut bacterial taxa were reported, thus providing new potential treatment approaches [73]. Polyphenol metabolites like gallic acid derivatives have also demonstrated neuroprotective targets [74]. These findings provide further impetus to look into novel pathways and therapeutic approaches for treating numerous neurodegenerative diseases.

## Conclusion

Gut-derived metabolites play a significant role in various functions of the host. They modulate the immune system by influencing the levels of several pro-inflammatory cytokines; they can exert neuro-inhibitory and neuro-excitatory effects; they possess anti-oxidant properties and influence epigenetic mechanisms. Factors such as the permeability of the gut and mucus secretion levels determine the type of bacteria present and the metabolites they produce, so when dysbiosis occurs, these factors are altered, which in turn change the levels of metabolites produced by the bacteria, thereby exerting beneficial or detrimental effects on the host. For example, supplementing metabolites like SCFAs has been shown to alleviate symptoms related to neurodegenerative disorders, such as inducing neurogenesis, preventing neural death, and reducing oxidative stress. The exact underlying mechanisms of most of these metabolites are not understood; however, evidence of their effects on the brain has been reported in multiple studies. Approaches such as modulating the levels of metabolites either by controlling dietary sources or supplementing them externally have been shown to demonstrate significant changes, be it ameliorating or exacerbating symptoms, making them promising targets for novel treatment approaches.

## Data Availability

Not applicable.
